# Concerns of Female Adolescents About Menarche and First Sexual Intercourse: Mixed Methods Analysis of Social Media Questions

**DOI:** 10.2196/13158

**Published:** 2019-06-04

**Authors:** Nikkie Saldivar Hodgson, Elad Yom-Tov, William F Strong, Priscilla L Flores, Giselle N Ricoy

**Affiliations:** 1 University of Texas - Rio Grande Valley Brownsville, TX United States; 2 Technion Haifa Israel; 3 Microsoft Research Herzeliya Israel; 4 School of Osteopathic Medicine University of the Incarnate Word San Antonio, TX United States

**Keywords:** menarche, sexual intercourse, social media, infodemiology, infoveillance

## Abstract

**Background:**

Adolescents use social media for information on medical and social aspects of maturation.

**Objective:**

The aim of this study was to investigate the concerns and information needs of adolescents regarding menarche and first sexual intercourse.

**Methods:**

Questions about menarche or first sexual intercourse were obtained from Yahoo Answers, a community-based social media question-and-answer website. A total of 1226 questions were analyzed. We focused on 123 question pairs made by users who asked questions on both topics and reported their ages at each. Quantitative and qualitative analyses were performed on these question pairs.

**Results:**

Qualitative analysis identified uncertainty as a significant theme for both menarche and first intercourse. Quantitative analysis showed that uncertainty was expressed in 26% (13/50) of menarche questions and 14% (7/50) of intercourse questions. Lack of communication was expressed in 4% (2/50) of menarche questions, compared with 8% (4/50) of intercourse questions. Ages at menarche and at first sexual intercourse were correlated, with women reporting menarche at the age of 13 years or younger being 2.6 times more likely to experience first sexual intercourse before the age of 16 years (*P*<.001, chi-square test). Older age at menarche was associated with greater lack of communication with parents (analysis of variance, *P*=.002).

**Conclusions:**

The questions of adolescents on the topics of menarche and first sexual intercourse express anxiety and uncertainty and are associated with a lack of information and deficient communication with parents. The more normative and expected a behavior, the less these factors appear. Therefore, parents and educators should, to the extent possible, improve communication around these topics, especially when they occur at less typical ages.

## Introduction

### Background

Today’s generation of adolescents is the largest in human history. With a population of 1.8 billion, 10- to 24-year-olds now comprise over a quarter of the global population. Adolescents have been a neglected age group in health care owing to various socioeconomic, political, and cultural factors that may deprive this age group of information, services, and health care professionals who are age-appropriate, gender-appropriate, nonjudgmental, and supportive [1]. Adolescents’ search for care may be restricted owing to barriers such as fear, embarrassment, lack of knowledge, misinformation and myths, stigma, and shame [1]. As confidentiality regarding sexual and reproductive health (SRH) issues is a major concern to adolescents, they often turn to the internet for answers to their health-related questions [2]. Access to SRH information is vital as adolescents are significantly at risk for premature parenthood, serious injury and infections, mental disorders, and substance abuse [3]. According to Morris and Rushwan [1], “young people are reaching puberty earlier, often engaging in sexual activity at a younger age, and marrying later.” As a result, adolescents are sexually mature preceding marriage for a longer time than ever before, lending to the pivotal importance of adolescent SRH.

During adolescence, the adult self begins to emerge and one’s self-identity is born [4]. The physical, emotional, and psychosocial changes occurring at this stage prompt numerous SRH questions [2]. At this time, a child begins making decisions independently of the parents and begins to build the foundation for future health and well-being. These decisions regarding sexual attitudes, values, and risk-related behaviors can be influenced by parental modeling of honest, transparent communication and conveyance of sexual information. However, sexual communication between the parent and adolescent often does not take place and barriers such as judgment, misinformation, and low self-efficacy [5] arise. The aim of this study was to explore concerns of adolescents surrounding SRH as it relates to menarche, first sexual intercourse, and psychosocial environmental influence.

### Conceptual Framework for Adolescent Health

The onset of puberty has long been accepted as the starting point of adolescence [6]. Key social role transitions such as completion of education, commencement of employment, marriage, and child rearing have historically signaled the completion of adolescence and entry into adult life [6]. Improvements in childhood hygiene, nutrition, and health have resulted in downward trends in the age of onset of puberty. This trend has essentially ceased in the majority of high-income countries where the main age of menarche (used as a proxy marker for pubertal development) has largely stabilized at about 12 to 13 years [6]. At the same time, there has been a dramatic increase in the age at which adult social roles and responsibilities are being adopted. In most countries at present, adolescents are spending longer times obtaining an education and are marrying and having children later [7].

In this study, the intersection of biology and social context in adolescent SRH is examined as it influences health-related behaviors and health outcomes. An example of a health-related behavior is engagement in unsafe sexual activity. Adolescents make up a quarter of the sexually active population in the United States and acquire half of all sexually transmitted infections [5]. Adolescents are also at risk for unintentional pregnancy. These health behaviors have significant consequences for adolescent health, their future life determinants, and a long-term impact on their families and communities [5].

## Methods

### Data and Data Collection

We extracted, using a crawler, a sample of question pairs from Yahoo Answers [8], a community-based social media question-and-answer website, such that each pair was made by the same user (albeit at different times). We required that 1 question in the pair contained the words *first period* and a phrase that could indicate an age (ie, “I’m XX,” “XX year,” or “XX year old,” where XX are numbers between 0 and 30), and the second question in the pair contained the word *sex* and phrases that would indicate first intercourse (ie, *lose* or *lost* within 10 characters of the word *virgin*, *virginity*, *boyfriend*, or *first time*).

The extracted questions were independently labeled by 2 of the authors such that 100 questions were labeled by both, and the remaining questions by one of the authors. Each question was labeled as to whether the first question in a pair was related to menarche and separately whether the second question was related to first intercourse. In addition, for those questions labeled in the affirmative, the labelers extracted the age of menarche or first intercourse, if the asker provided it in the question. Agreement among the raters was estimated for the 100 questions labeled by both using Cohen kappa statistic [9].

After filtering, the questions were paired according to the anonymized user identifier provided by Yahoo Answers.

These data are particularly reliable because previous studies [10,11] showed that anonymity provides for honesty when matters of health are at stake. Many reliability studies have demonstrated that such data are particularly reliable because the subject wants credible information and advice about their problems and concerns [12].

This study was approved by the Institutional Review Board of the Technion.

### Analysis

A narrative approach was used to analyze the dataset to determine the distrust adolescent females have regarding parent-adolescent communication about menarche and first sexual intercourse. We subjected the content of all the questions and comments of each subject to a QSR NVivo analysis [13] to identify recurring themes within the dataset. Specifically, transcripts of the questions were coded to identify consistent repetition of relevant words, phrases, sentences, and sections. On the basis of this coding of the transcripts, categories were created and sorted into themes. Categories were then labeled on the basis of the connections between the codes that made them up.

The data were then subjected to quantitative analysis on the variables of menarche and first intercourse.

## Results

### Data

A total of 1226 questions were analyzed. Of those, 270 were judged to discuss menarche and 147 were judged to discuss first intercourse. A subset of 123 users asked questions in both the menarche and first intercourse sets and reported ages therein.

Cohen kappa agreement among raters for whether the first posting was menarche-related was 0.72 (*P*<.001). The agreement for whether the second posting was about first intercourse was 0.87 (*P*<.001). These values are considered very high.

### Qualitative Analysis

**Figure 1:**
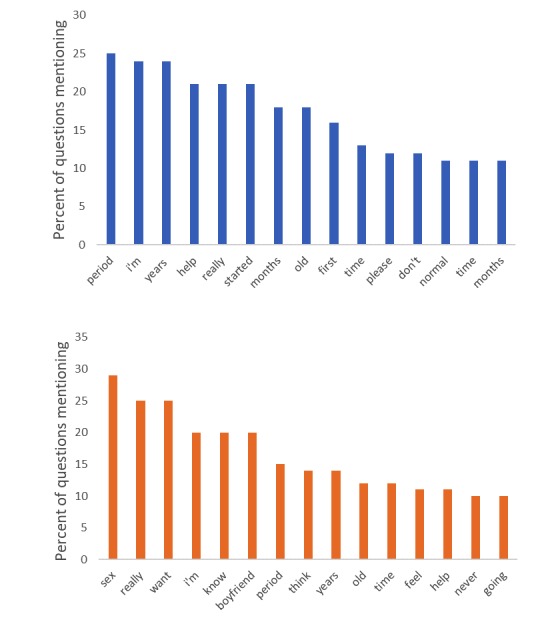
The most popular words for menarche (top) and first intercourse (bottom). All words that appeared 10 or more times, excluding stopwords, are shown.

Figure 1 shows the most popular words in menarche and, separately, first intercourse questions. In the qualitative analysis, we identified 1 significant theme from the participants’ narratives as it relates to menarche and first sexual intercourse: uncertainty. This theme is supported by the following subthemes: lack of accurate information about their own growth and development and a lack of communication with family and peers. The results led to insights for fostering a transparent, honest climate for sexual communication.

#### Uncertainty

This theme is based on the presence of anxiety, worry, and fear about becoming fully developed, a full-fledged adult, and a woman. These changes during adolescence create confusion about what is normal as it relates to menarche and first sexual intercourse. The following are examples of questions asked on the community-based question-and-answer website demonstrating the theme of uncertainty used in our dataset:

1. “I’m going to a sleepover tonight but I’m on my period. I’m 13. I don’t know what to do because I don’t want people to find out. Help!!”
2. “I’m 13 years old and 10 months and my period came from 10 months and until now I don't have any period. I’m a virgin. I’ll be going to the GYNO and I’m little bit scared but will the doctor examine me? If yes, what should I do, like shave, wipe the discharge? Please help me. Take your time answering, I’m scared.”
3. “Ok so I’m 13 and I really want to have sex, is 13 ok? I’m really confused. Can you help me? I am scared and confused. I know I’m young, but I’m like obsessed with it. Can you help me?”

#### Lack of Information and Lack of Communication

In the narratives, there is a lack of clarity about the experience of menstruation and sexual desire/activity and a lack of communication for support and knowledge from parents and peers, which in turn creates myths and misconceptions. The following are examples of questions asked on the community-based question-and-answer website demonstrating the subtheme of lack of information and lack of communication used in our dataset:

1. “Okay I live with my dad, and like NEVER see my mom, or my grandparents or my aunt, and I don’t really feel comfortable telling my dad or either my grandparents are worse! I don’t feel comfortable confronting my dad face to face, and emailing him or writing a note to him, seems kind of stupid (no defense), but how do I tell my dad I started my period? Next month will be my 4th period, please help. Like 3 years ago (btw I’m 14 now) he got my aunt to buy me some pads and talk to me about it. Please give me hints and/ or tips on how to tell him or even your own personal stories! Also I’m really shy so I don’t feel comfortable talking to any friends moms about it! BUT PLEASE HELP ME!”
2. “Help!!!!!!!! I'm 11 yr. 12 in August, I have no idea what to do. I told my mom, but she said it’s kind of early for me... I'm the only one in my class, who has started and how am I supposed to hide it? I just take it..Help!!! I need somebody, I feel really alone. I know if I tell one of my 3 sisters they are going to tease me and say awwwww you are growing up. I just have no idea what to do!!! OMG!”
3. “I’m 18 and my mom made me an appointment for the gyno because I have irregular periods...at the time she made it I had never had sex before...last night was my first time...she filled out the sheet and everything saying no I’m not sexually active and all that...I’m not telling her that I had sex last night. I do not plan on having sex again anytime soon considering it was a big mistake last night. Since it was my first time and I’m not going to be doing it all the time. Now do I have to tell the gyno I have had sex? If so, should I just leave the sheet checked no and then tell them I filled that out before it happened?”

### Quantitative Analysis

**Figure 2:**
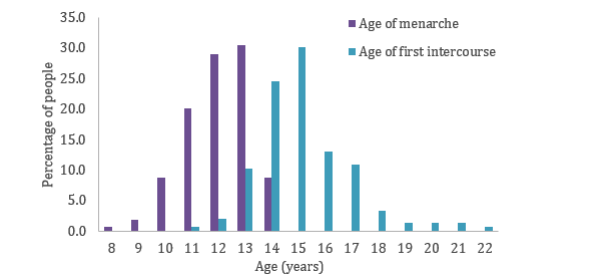
Distribution of age at menarche and age at first intercourse as reported in the questions.

A total of 270 women reported their age in their menarche question, and 147 women reported their age in their first intercourse question. The distribution of ages is shown in Figure 2. The agreement between the cumulative distribution of reported age of menarche and the distribution reported in the literature [14] is 0.94 (*P<*.001). The agreement for first intercourse (compared with a study by Martinez and Abma [15]) is 0.95 (*P*=.01).

**Figure 3:**
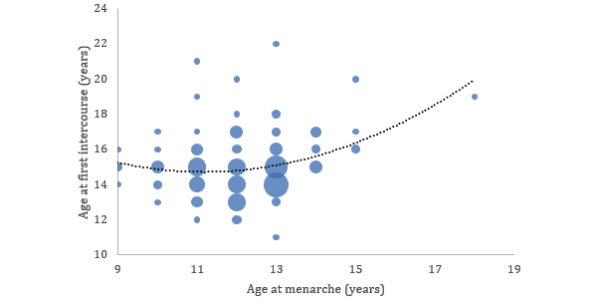
Correlation between the age of menarche and the age of first intercourse (n=123). The dotted line shows a fit of a second-order polynomial with *R*^2^=0.1.

Figure 3 shows the correlation between the reported age of menarche and the age at first intercourse. As the figure shows, there was no association between the 2 ages for women whose age at menarche was 12 years or lower. However, when the age at menarche was higher, so was the age at first intercourse. Specifically, we found that women reporting menarche at the age of 13 years or younger were 2.6 times more likely to experience first sexual intercourse before the age of 16 years (*P*<.001, chi-square test). This finding is compared with literature that identifies adolescent females experiencing early menarche as twice as likely to have had intercourse by the age of 16 years than adolescent females with late menarche [16].

Following the themes identified in the qualitative research, we coded 50 question pairs for uncertainty about future effects, lack of information, and lack of communication with parents. Strikingly, 86% (43/50) of menarche questions and 44% (22/50) of first intercourse questions contained requests for information. Uncertainty was expressed in 26% (13/50) of menarche questions and 14% (7/50) of intercourse questions, and lack of communication was expressed in 4% (2/50) of menarche questions compared with 8% (4/50) of intercourse questions. An analysis of variance model found statistically significant interactions only between age of menarche and lack of communication (with higher ages reporting more communication problems; *P*=.002).

## Discussion

This study examined the concerns and information needs of adolescents regarding menarche and first sexual intercourse as evident from questions on a social question-and-answer website, Yahoo Answers. Although online data have been used to study aspects of population health and especially health communication [17], we suggest that the use of online data, particularly social media, to study aspects of younger adult health is advantageous because of these young adults make extensive use of such media, particularly for sensitive topics [11], such as sexual health.

This study’s quantitative research shows that the older the adolescent female was at menarche, the older she was at first intercourse, as demonstrated in the previous literature [16]. The age at menarche can be attributed to additive genetic effects, dominance effects, and unique environmental effects, including, but certainly not limited to, *in utero*, population health, socioeconomic status, and psychosocial factors [18]. Our qualitative research explored the psychosocial environmental influences and found that the most significant and recurring theme regarding menarche and first sexual intercourse in questions asked by adolescent females in our dataset was uncertainty. The recurring, significant subthemes were lack of information and lack of communication. We can determine that adolescents are uncertain about what is normal and the impact that their behaviors (menarche and sexual intercourse) will have on their livelihood, their families’ livelihood, and their communities’ livelihood. This uncertainty, lack of information, and lack of communication is influenced by the adolescent’s sexual socialization via their relationship and level of communication with parents, peers, and health care providers. Educators also play an important role in communicating information [19]. Though in many cases they are challenged in developing programs to effectively help adolescents to understand their uncertainties, surveys show that educators have become a more important source of information in recent years [20].

As a child enters adolescence, he or she is faced with all sorts of new information about sex, reproduction, culture, and societal expectations. Parents are vital agents of sexual socialization for children and adolescents. As per Bronfenbrenner’s ecological systems theory [21], qualities of an adolescent’s environment (including the family system) will reciprocally and dynamically influence development. It is generally considered that parent-adolescent communication is associated with more responsible SRH among adolescents. According to Hutchinson et al [22], parents who communicate with their children about sex can positively influence sexual behaviors. Parent-adolescent sexual communication, especially mother-adolescent conversations, plays a protective role in more responsible SRH behaviors among adolescents that is more significant for female adolescents [5,23]. For example, 1 study identified “design and implementation of family-based approaches to improve parent-adolescent sexual risk communication as one means of reducing HIV-related sexual risk behaviors among inner-city adolescent females” [22]. Although adolescents may attempt to get their SRH information from other means (ie, peers or the internet), it is important for parents to discern that information, falsify the myths, and confirm the facts. In addition, it is important for the conversation about SRH to begin early and continue into emerging adulthood in the early twenties, where the riskiest behavior is likely to take place [24]. According to the literature [24], the parent-adolescent dialogue about SRH is one that, if begun early, is normative and healthy. Often, parents are inclined to shy away from discussing SRH because they are not comfortable doing so [25] and tend to delay discussions with their child until they believe they are already engaging in sexual behavior. Parents who do not take the time to enhance their own information and skills on SRH, are likely to disseminate inaccurate information resulting in less successful parent-adolescent communication and less positive impact on their child’s SRH [26]. It is important for parents to communicate that they value their child’s or adolescent’s interest and opinions about SRH and be comfortable empowering their child or adolescent with SRH information, be it from themselves or a health care provider.

We found that menarche questions are primarily a request for information about current experiences, with less of an emphasis on future effects. This may be partly due to younger women finding it easier to communicate with parents on issues considered *normal* at their age, compared with teenagers who are older at the time of menarche. Many first sexual intercourse questions request information. However, here lack of communication is greater, reflecting an uneasiness to reveal highly personal behaviors to parents [27].

The media is also an important agent of socialization for adolescents and can also give adolescents contradictory messages. As adolescents are concerned about confidentiality regarding SRH, the internet serves as an accessible, anonymous, and interactive modality to explore sensitive or taboo topics that they may not feel comfortable asking parents or health care providers about. For example, the community-based question-and-answer website this study used to collect data is one in which any user of the website can supply *answers*. The major potential drawback to this mode of information is potentially inaccurate information and abusive language or content [2]. Religious beliefs, race, ethnicity, and education are other agents of sexual socialization within the family that can influence an adolescent’s sexual self-identity [28].

The barriers adolescents face (ie, societal norms, confidentiality concerns, and embarrassment ) accessing SRH care may explain why nearly one-fourth of young people say that they have not discussed sexual topics with a parent [5]. We believe that strategies, programs, and policies that aim to create a more supportive environment for adolescents to foster safer, more accessible, and informative care should be developed. Overall, 1 study showed that adolescents describe a beneficial health care provider to be friendly, which can be improved via service provider training and assured confidentiality [29]. Interventions can also be aimed at the parents of adolescents so that they learn what SRH information to discuss with their adolescents and how to convey that information to their adolescents. These interventions can help parents view themselves as valuable sources of information who can help shape their children’s sexual socialization and acquire information and skills that make them more effective communicators [30]. Parents can model transparent, honest communication by being an approachable and attentive parent, including becoming aware of verbal and nonverbal behavior, tone of voice, content of verbalizations, warmth, and respect, which encourage more frequent and spontaneous conversations about SRH. As other literature divulges, further research on prevention of risky adolescent SRH should be aimed to inform programs and policies that target younger, at-risk adolescents [31]. Health care professionals and parents should recognize, consider, and invest in sources of sexual education to promote SRH responsibility in adolescents as parent-adolescent communication is a sexual health determinant and can influence the uncertainty that a female adolescent encounters at menarche and sexual maturity.

## Abbreviations

SRH: sexual and reproductive health
